# Synchronising anti-predator behavior in the red flour beetle *Tribolium castaneum*

**DOI:** 10.1093/beheco/araf013

**Published:** 2025-02-18

**Authors:** Kentarou Matsumura

**Affiliations:** Department of General Systems Studies, Graduate School of Arts and Sciences, The University of Tokyo, 3-8-1, Komaba, Meguro, Tokyo, 153-8902, Japan

**Keywords:** anti-predator strategies, death feigning, thanatosis, harding behavior, *Tribolium castaneum*

## Abstract

In many animals, a phenomenon is often observed in which behavior depends on population density and many individuals within the group synchronize their state of behavior to some extent, and theoretical studies have suggested that this synchronization phenomenon is adaptive for predation avoidance. Moreover, death-feigning behavior (DF) has been observed as an anti-predator strategy in many animals. There are large individual differences in the duration of DF, and the optimal duration of DF often varies depending on the situation. Therefore, although it is expected that prey may synchronize with others around them for an optimal DF duration, there are few experimental studies testing this hypothesis. This study investigated whether DF duration varies with and without other individuals, and whether it synchronized with the DF duration of other individuals, in the red flour beetle *Tribolium castaneum*. This study used populations with genetically longer (L-population) and shorter (S-population) DF duration and measured DF duration when maintained alone and cohabitated with individuals from the L- and S-populations, respectively. The results showed that the DF duration of individuals living alone increased significantly compared to pretreatment. Moreover, individuals that cohabitated with S populations were significantly shorter after cohabitation, but the presence of the L population did not cause any changes in how individuals synchronized their activities. When many individuals had shorter DF durations, DF was synchronized towards shorter durations. This is the first study to illustrate the synchronization of anti-predator behavior in terms of DF behavior.

## Introduction

In many animals, including humans, a phenomenon where many individuals within the group synchronize their behavioral state to some extent is often observed (Parrish and Hamner 1997; Ruxton 2002; Slater et al. 2003). For example, starlings exhibit behaviors that make many individuals fly in groups (Cresswell 1994; Ruxton 2002; Roth et al. 2006). In addition, the illumination of fireflies has been observed to synchronize with other individuals (Buck and Buck 1968). This phenomenon is suggested to be important in social or predation avoidance (Emlen 1952; Okubo 1986; Buhl et al. 2006). Indeed, some theoretical studies have suggested adaptive significance of this phenomenon (Ajraldi et al. 2011; Matia and Alam 2013), and several empirical studies have investigated synchronous behaviors (reviewed in Duranton and Gaunet 2016).

In many animals, because exposure to predator attack leads to injury and death, adaptive antipredator strategies in prey animals evolve rapidly (Edmunds 1974; Lima and Dill 1990; Sih 1992; Lima 1998; Ruxton et al. 2019). In contrast, because the types and numbers of predators often change (Laurila et al. 2008; Díaz et al. 2013; Zhang et al. 2020), a prey’s antipredator behavior cannot be completely genetically controlled, and individuals exhibiting adaptive plasticity in response to environmental fluctuations. For example, it has been shown that in leopard frog tadpoles (*Rana pipiens*), predator avoidance behavior and activity can be plastically altered by predator cues and injured conspecific cues (Schoeppner and Relyea 2009). Furthermore, prey density, not only predators, is also important in predation avoidance. For example, the larger the group of prey animals is, the smaller the chance that any particular individual will be attacked by predators. This is how individuals gain protection from predators by joining a group—a simple “dilution” effect (Hamilton 1971; Foster and Treherne 1981; Turner and Pitcher 1986). Moreover, even in a large group, if their behavior differs from that of other individuals, they make it easier for predators to find them. Predation risk can influence prey population density. Additionally, the behavior of other individuals within the population can also be affected. In response to these combined factors, prey with the ability to adapt their antipredator behavior (plasticity) may modify their strategies to avoid predators (Schoeppner and Relyea 2009). However, there are few studies that have investigated synchronize to other individuals with a focus on predation avoidance behavior.

Death-feigning behavior (DF), which a state of immobility as a response to stimulus is an adaptive antipredator strategy across a wide range of animal taxa (Miyatake et al. 2004; Humphreys and Ruxton 2018; Sakai 2021). The sudden immobility of the prey when the risk of predation is high causes the predator to lose sight of the prey or lose interest in the prey. Some empirical studies revealed that adaptive significances of DF (Humphreys and Ruxton 2018; Sakai 2021). For instance, red flour beetles *Tribolium castaneum* that feign death for longer periods have a higher survival rate when encountering jumping spider *Hasarius adansoni* as predators, compared to those with shorter DF durations (Miyatake et al. 2004). Thus, it is suggested that the DF duration of prey evolves in the direction of longer duration under high predation pressure. On the other hand, individuals with long DF are more vulnerable to physical stress than short DF (Kiyotake et al. 2014). Moreover, individuals with long DF are less active and thus have fewer mating opportunities (Nakayama and Miyatake 2010a, 2010b). A previous study revealed that population that suffered predation pressure indicated longer DF duration than population was reared in the absence of a predator in field (Konishi et al. 2020). Therefore, the optimum DF duration is expected to vary depending on environmental conditions, such that longer DF is adaptive under high predation pressure and shorter DF is adaptive under low predation pressure.

Although DF has been revealed to be an adaptive antipredator behavior for prey, its effectiveness often depends on the existence of other conspecific individuals. Previous study revealed that *T. castaneum* avoidance of predation was higher when conspecific individuals were present in the vicinity than when conspecifics were absent, even for individuals with long DF (Miyatake et al. 2009). This is because during long DF, other conspecific individuals that do not exhibit DF are attacked by predators instead. That is, it has been suggested that the DF strategy increases its effect at the expense of other conspecific individuals, which is called the “selfish-prey hypothesis” (Miyatake et al. 2009). A prey feigned death at a high prey population density increases the likelihood that a predator will shift its interest to another prey. That is, if the prey adopts a DF under high population density of the prey, the predator will change its target to another prey in a shorter time. Therefore, it can be inferred that the predation avoidance success of focal prey is high even if the DF duration of focal prey is short under the condition of high population density of prey. Conversely, a predator may remain interested in its prey even if the prey becomes death feigning long duration when the population density of the prey is low (ie no other individuals of the same species are around). Therefore, the optimal duration of DF may depend on their population density, and it is predicted that there is negative relationship between DF duration and population density in the prey.

Furthermore, not only the presence or absence of surrounding conspecific individuals but also the DF duration of the conspecific individuals may affect to predation avoidance. Here, I have proposed the following hypothesis: if the DF duration of surrounding conspecific individuals is short, the optimal DF duration is relatively short because these conspecific individuals may become the sacrifice. In contrast, if surrounding conspecifics have a long DF duration, the optimal DF duration is expected to be relatively long. That is, when an individual is in an environment where there are many individuals with long DF, the individual will have more possibility to be first attacked by the predator. That is, individuals exhibit long DF duration in the environment. Therefore, the DF duration of the prey synchronizes with the DF duration of conspecific individuals.

**Fig. 1.**
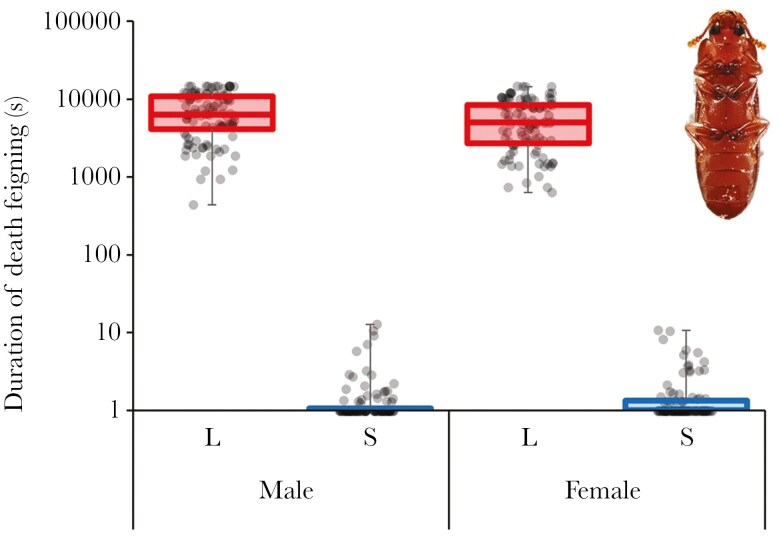
*Tribolium castaneum* feigning death (upper right), and DF duration of L- (red) and S- (blue) populations.

This study examined how the presence or absence of conspecific individuals and the DF duration of conspecific individuals in the surrounding area affect the DF behavior in *T. castaneum*. In a previous study (Miyatake et al. 2004), populations with genetically longer (L-population) and shorter (S-population) DF duration have been established by artificial selection for DF duration in *T. castaneum* (Fig. 1). This previous study indicated that DF duration has a genetic basis in *T. castaneum*. On the other hand, in another previous study using this L strain, it was found that the L strain beetles presented with the aggregation pheromone shortened DF duration (Ishikawa et al. 2023). Thus, DF duration is genetically controlled to some extent, but that it changes plastically according to the situation. Using the selected populations, this study compared the DF behavior of individuals in the presence or absence of conspecific individuals and in the presence of L- or S-populations.

## Materials and methods

### Insect and culture

A stock culture of *T. castaneum*, in laboratories for more than 30 yr, was used in this study. The beetles were reared on a mixture of wholemeal enriched with yeast in an incubator maintained at 25 °C under a 16:8 h (light:dark) photoperiod (lights on at 7:00; lights off at 23:00). Distinct morphological characters of the abdomen were used in sex determination of beetles at the pupal stage. Then, single-sex groups were stored in a dish (diameter 90 mm, height 15 mm) until experiments were performed. The focal individuals in each treatment were randomly collected from this stock culture.

### Selected populations for duration of death feigning

L-population with long DF duration and S-population with short DF duration in *T. castaneum* were established by a previous study (Miyatake et al. 2004), and these selection regimes have been maintained for over 30 generations (Matsumura and Miyatake 2018). More details of this selection were described in previous studies (Miyatake et al. 2004; Matsumura and Miyatake 2018). Although approximately 20 yr have passed since the selected populations were established, large differences in DF duration between the L- and S-populations have been maintained (Fig. 1).

### Experimental design

**Fig. 2.**
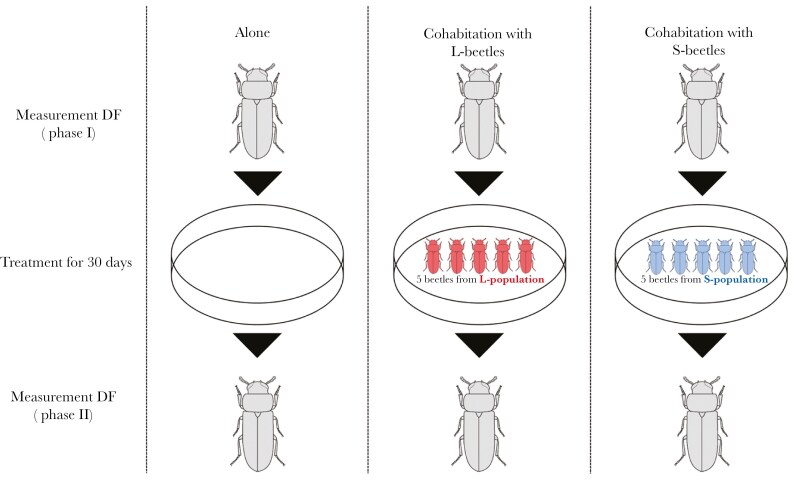
Experimental design of this study.

Experimental design of this study is shown in Fig. 2. Firstly, I measured DF duration (phase I) according to a previously described method (Miyatake et al. 2004). Virgin beetles (21 to 35 d old) were used. The beetle body was stimulated by a woody stick. If the beetle showed immobility, the duration of this behavior (until the beetle moved) was recorded by stopwatch. If the beetle did not show DF behavior by stimuli, the stimulus was repeated up to three times for each beetle.

On the next day of measurements of DF behavior, each beetle from the stock culture was put into a plastic container (height 15 mm, width 10 mm) with food, and three treatments were performed (alone; male: *n* = 24, female: *n* = 24, cohabitation with L; male: *n* = 38, female: n = 48, cohabitation with S; male: *n* = 38, female: *n *= 48). In the alone treatment, each beetle kept alone for an additional day. In the cohabitation treatment, a beetle (focal beetle) was cohabitated with five beetles from L- or S-populations in the plastic container. There was no significantly difference in DF duration among the treatments at phase I (*P* = 0.457; Table S1). To identify focal and cohabitant beetles, the elytra of *T. castaneum* was marked with paint marker following to methods described in previous studies (Matsumura and Miyatake 2015; Matsumura et al. 2021). To eliminate the effect of marking on results, I marked cohabitants rather than focal beetles. All containers were kept for 30 d in an incubator maintained at 25 °C and 16:8 h photoperiod. Each container was filled with 1.0 g of food, enough for six beetles to live for 1 mo. To avoid influences of copulation, beetles were separated by sex in each treatment. After treatment, measurements of DF duration for focal beetles were repeated (phase II).

### Statistical analysis

To test the effects of treatment and cohabitated beetles on the DF duration, generalized linear mixed models (GLMMs) with gamma distribution (Ishikawa et al. 2023; Matsumura and Miyatake 2023), phase, treatment, sex, and interactions of these factors as explanatory variables, beetle ID as a random effect were used for all DF duration data. The GLMM with same model was also used to analysis for DF frequency (binary data of whether or not shown DF). When a significant effect of interaction among factors was found, multiple comparisons using Bonferroni correction were performed. All analyses were conducted using R version 4.1.0 (RCoreTeam 2017) and statistical packages *lme4* (Bates et al. 2015) and *car* (Fox and Weisberg 2018).

## Results

**Fig. 3.**
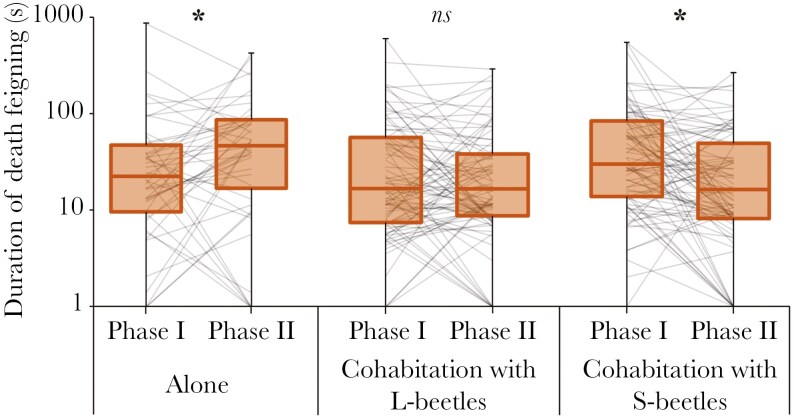
DF duration of each treatment in phase I and II: (*a*) alone, (*b*) cohabitation with L-beetles, and (*c*) cohabitation with S-beetles. The asterisk shows significant differences between phase I and II (*ns* shows no significant difference).

The proportion of individuals that showed DF at phase I was 93.8% (45/48) in alone treatment, 96.5% (83/86) in cohabite with L, and 97.7% (84/86) in cohabite with S. The proportion of individuals showing DF at phase II was 89.6% (43/48) in alone, 91.7% (79/86) in cohabited with L, and 87.2% (75/86) in cohabited with S. Figure 3 shows the DF duration in each treatment of phase I and II. Table 1 shows the results of GLMM for DF duration both phase I and II. In results of GLMM for all data, although the effects of phase and treatment were not significant, interaction between phase and treatment showed a significant effect on DF duration (Table 1, Table S2). The results of post hoc tests with Bonferroni correction for this GLMM result showed significant differences between the phases in the “alone” and the “cohabitated with S-beetles” (Fig. 3). In the “alone” treatment, DF duration was significantly longer in phase (2) than in phase (1). In the “cohabitated with S-beetles,” DF duration was significantly shorter in phase (2) than in phase (1). There was no significant difference in the DF duration between the phases in “cohabitated with L-beetles” treatment. There were significant effects of sex and interaction between phase and sex on DF duration (Table 1). Male beetles showed significantly longer DF duration than females in both phase I and II (Fig. S1; Table 1; Table S1), and the intensity of decrease in DF duration was larger in males than females regardless of treatments (Fig. S1; Table 1). There was no significant difference between phase I and phase II in the proportion of individuals showing DF (Table S1-3).

**Table 1.** Results of GLMM for DF duration.

Factor	*d.f.*	*χ* ^2^	*P*
Phase	1	1.93	0.1648
Treatment	2	2.83	0.2425
Sex	1	18.82	**< 0.0001**
Phase*treatment	2	29.23	**< 0.0001**
Phase*sex	1	20.93	**< 0.0001**
Treatment*sex	2	1.19	0.5515
Phase*treatment*sex	2	4.69	0.0957
Error	414		

## Discussion

This study examined the influence of the presence of conspecific individuals and the characteristics of cohabitated individuals behavior on the DF, which as anti-predator behavior in *T. castaneum.* Present results indicated significant effects of this treatment to DF duration in focal beetle. The DF duration of an individual varied long under a solitary environment with no other individuals around (ie alone treatment). Population density often exhibits temporal change in many animals (Gaillard et al. 2000). It is therefore possible that the predator avoidance behavior of prey may evolve to change plasticly in response to ever-changing population density. This study result support this hypothesis, as the present study revealed that the DF of *T. castaneum* can change plasticly in response to population density.

Moreover, the DF of individuals cohabited with S-beetles changed shorter. These results suggest that the predator avoidance behavior varies with presence or absence of surrounding conspecifics and synchronizes with surrounding conspecifics in *T. castaneum*. If there are many individuals around own with shorter DF duration, own predation avoidance rate may be higher. However, because excessive length of DF is often costly (Nakayama and Miyatake 2010a, 2010b), it may be more adaptive to vary short DF duration in such an environment. On the other hand, because risk of predation for individuals with short DF is high in solitary environments, it may be more adaptive to plastically alter DF to longer in this environment. These predictions were supported by this study. These results are important because this is the first study to reveal that DF duration is changed and synchronized.

However, when other individuals in the population have a longer DF duration, there was no significant change in the DF duration of the individuals. Because prey with a shorter DF duration than other prey in the population is at higher risk of predation (Miyatake et al. 2009), it is expected that the DF duration of beetles would be longer in populations with longer DF duration. However, although DF duration changed longer in the solitary treatment, DF duration did not change by cohabitation with L-beetles. The current results suggested that *T. castaneum* does not respond to the long DF duration of surrounding individuals. Moreover, there is a possibility that this species cannot recognize the DF duration of other individuals around it.

One reason why DF duration did not increase after cohabitation with L beetles may be the trade-off between DF duration and locomotor activity. Previous studies revealed that L-beetles exhibited lower activity whereas S-beetles exhibited higher activity in *T. castaneum* (Miyatake et al. 2008; Matsumura et al. 2016). Therefore, it is expected that when S-populations are around, focal individuals have more contact with other individuals. Therefore, subjects might have perceived that there were many other individuals around them. In contrast, because beetles from L-populations had lower locomotor activity, subjects had less contact with other individuals and might have perceived that the number of other individuals around the focal individual was small. Thus, DF duration might not have changed. On the other hand, because it is expected that they had at least a few opportunities to encounter L-beetles compared with the alone beetles, the change that increased DF duration such as alone beetles may not be occurred. That is, there is a possibility that the DF duration fluctuates simply due to the physical contact frequency with other individuals. Indeed, in a previous study used sweet potato weevil (*Cylas formicarius*), the possibility that contact with other conspecific individuals may shorten death-feigning duration was mentioned, but detailed data were not presented (Miyatake 2001; Miyatake et al. 2008). To investigate this possibility, it is important to observe the behavior during cohabitation treatments. However, because it is difficult to observe the behavior of beetles in wheat flour, which is the food of this species, I did not observe it in this study. It is also necessary to provide only beetle contact stimuli, eliminating odors and visual information. Furthermore, it would be useful to provide artificial stimuli that mimic contact stimuli between beetles, but it was difficult to conduct these experiments in this study. This presents a future challenge of investigate the effect of contact frequency with other individuals on the DF in *T. castaneum*. Alternatively, because the DF duration of the L-beetle used in this study was extremely long, as exceeding 1 h (Fig. 1), the focal beetle may had exceeded the range of recognition of length of the DF.

In the present study, males showed clearer responses to cohabitation treatments than females (Fig. S1). The tradeoff cost of long DF may large in males. For example, males with long DF had reduced mating success than short DF males in *T. castaneum* (Nakayama and Miyatake 2010b). This suggests that males with extended DF behavior experience reduced fitness success. Therefore, if the mating success is reduced due to the extension of DF duration and not by additional characteristics of L-populations, minimizing DF duration when not under predation pressure would be advantageous when possible. Conversely, if females do not exhibit a similar decline in mating success with prolonged DF, it would be beneficial for them to maintain this behavior to avoid any potential predator. Although there are known cases in which females can benefit directly and indirectly from multiple copulations (Jennions and Petrie 2000), in most cases, females that can receive the sperm needed to fertilize all eggs in a single copulation may not need to spend the time to copulate with increased predation risk (Arnqvist and Rowe 2005). Moreover, the present results may be influenced by pheromones. A previous study (Ishikawa et al. 2023) revealed that *T. castaneum* that sensed aggregation pheromones released only by males shortened their DF duration. This result indicate that males may have been exposed to pheromones released by their cohabitants, which may have shortened their DF duration. However, because a previous study (Matsumura and Miyatake 2019) reported that the amount of pheromone released by males did not differ between L and S populations, the effect of pheromone alone cannot fully explain the result that DF duration was decreased when cohabitate with S-population. It would be interesting to examine the DF duration when females with cohabitants were exposed to pheromones.

Previous studies have suggested that DF duration is strongly associated with dopamine levels in *T. castaneum* (Miyatake et al. 2008; Nishi et al. 2010; Uchiyama et al. 2019). Therefore, it is also interesting to examine the influence of physiological factors such as dopamine levels in reducing the DF duration via cohabitation with S-beetles.

The effect of the DF of surrounding conspecifics on predation avoidance may vary depending on the species of predator. Foraging types of predator are broadly divided into two types: exploratory predators, such as jumping spiders, which recognize prey by sight, and sit-and-wait predators, such as assassin bug, which recognize prey by vibration. Predation experiments in previous studies showed that L strain beetles with low activity exhibited higher survival rate when they were cohabited with an assassin bug (Asakura et al. 2022; Matsumura and Miyatake 2022). However, if the assassin bug recognized L strain beetle as prey, the survival rate was significantly decreased even if the beetle continued DF for a long time (Asakura et al. 2022; Matsumura and Miyatake 2022). This suggests that DF contributes to the avoidance of predation against a predator that relies on visual foraging, whereas that DF does not contribute to the avoidance of predation against a predator that does not rely on visual foraging (Asakura et al. 2022; Matsumura and Miyatake 2022). Low activity against sit-and-wait predators may be effective in avoiding predation (Matsumura and Miyatake 2015; Asakura et al. 2022). Thus, in environments with sit-and-wait predators, plastically changes in activity may be effective in avoiding predation. It is a future task to investigate the effect of conspecific individuals focusing on activity.

The treatment period in this study was 30 d; it considered the effect of density during the developmental period, as the species has a developmental period of ~30 d (Sokoloff 1974). Population density was not controlled during development. In any case, the point is that 30 d was enough to bring about a change in DF behavior. However, future research is needed to resolve questions about how many days later this change occurs and whether the amount of change in pretending to be dead varies with the length of the treatment period. Furthermore, in this study, density treatment was carried out so that five individuals were placed in a 10 mm container. It is important to examine the effects of density difference on DF behavior.

This effect may be selfish plasticity which increases the survival rate of the self by synchronizing to the behavior of the majority individual which exists in the circumference. In this study, I used beetles with a genetic based DF duration, but in the field, the DF duration varies greatly among individuals (Matsumura 2021; Matsumura and Miyatake 2023), which may cause them to vary their DF duration plastically. Within a population, each act in synchronize with the surrounding individuals, and DF duration may also be in synchronize within the population (at least in the short direction). In addition, although a longer DF duration is advantageous under high predation pressure, the DF duration may be synchronized to a shorter direction as predation pressure decreases, and this synchronized DF duration is expected to vary temporally with environmental changes. Finally, DF is a common predation avoidance behavior that has been identified not only in insects but also in a wide range of animal taxa, including mammals, birds, reptiles, amphibians and fish. Although this study examined only *T. castaneum*, the findings are may be common in other animal species. Investigation of the behavioral synchronization in DF in other animal species is important to behavioral ecology.

## Supplementary Material

araf013_suppl_Supplementary_Material

## Data Availability

Analyses reported in this article can be reproduced using the data provided by Matsumura (2025).
