# Supplementary material for: Synchronising anti-predator behavior in the red flour beetle Tribolium castaneum
Source: Behav Ecol. 2025 Feb 18;36(2):araf013. doi: 10.1093/beheco/araf013 (PMC11894372; doi:10.1093/beheco/araf013)
Supplement: araf013_suppl_Supplementary_Material [file araf013_suppl_supplementary_material.docx]

**Supplementary information**


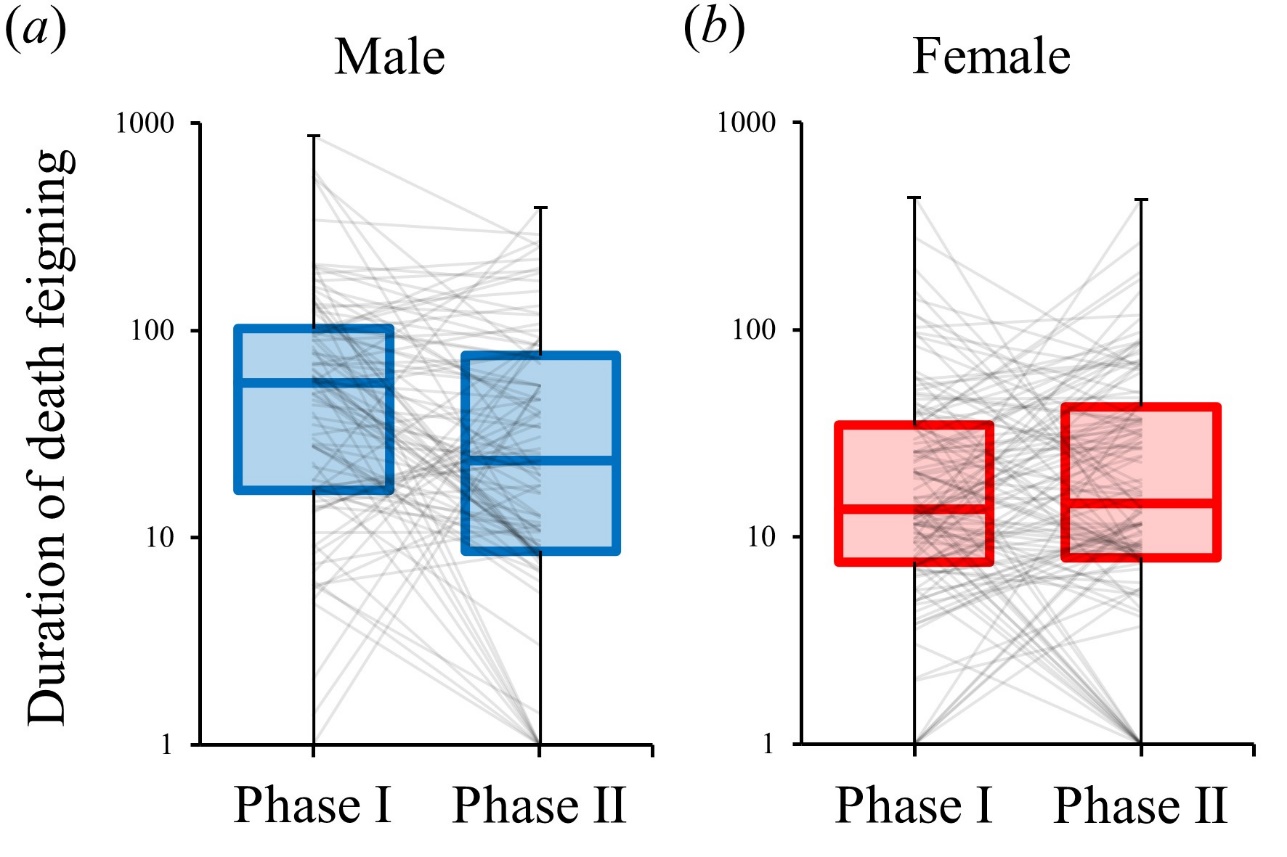


**Figure S1**. DF duration of male and female in phase Ⅰ and Ⅱ (regardless of treatments). There are significantly differences of sex and interaction between sex and phase (see Table 1).

**Table S1**. Result of GLM for duration and frequency of DF in phase Ⅰ. In this analysis, treatment, sex, and interactions between treatment and sex as explanatory variables were used for DF duration data in phase I. The GLM with same model was also used to analysis for DF frequency. I used chi-square test for each significance test. All analyses were conducted using R version 4.1.0 (RCoreTeam, 2017) and statistical packages lme4 (Bates et al., 2015) and car (Fox and Weisberg, 2018).

| Death feigning | Factor | *d.f.* | *χ*^2^ | *p* |
| --- | --- | --- | --- | --- |
| Duration | Population of cohabited beetle | 2 | 1.57 | 0.4566 |
|  | Sex | 1 | 21.7 | **< 0.0001** |
|  | Population of cohabited beetle*sex | 2 | 2.56 | 0.2778 |
|  | Error | 214 |  |  |
| Frequency | Population of cohabited beetle | 2 | 1.41 | 0.4953 |
|  | Sex | 1 | 1.62 | 0.2025 |
|  | Population of cohabited beetle*sex | 2 | 2.35 | 0.3088 |
|  | Error | 214 |  |  |

**Table S2**. Random effects of GLMMs for duration and frequency of DF in phase Ⅱ.

| DF | Group | Variance | SD |
| --- | --- | --- | --- |
| Duration | ID | 0.7399 | 0.8602 |
| Frequency | ID | 2.54E-13 | 5.04E-07 |

**Table S3**. Results of GLMM for frequency of DF.

| Factor | *d.f.* | *χ*^2^ | *p* |
| --- | --- | --- | --- |
| Treatment | 1 | 1.31 | 0.253 |
| Population of cohabited beetle | 2 | 0.37 | 0.8315 |
| Sex | 1 | 0.06 | 0.8059 |
| Treatment*population of cohabited beetle | 2 | 0.88 | 0.6425 |
| Treatment*sex | 1 | 0.06 | 0.8135 |
| Population of cohabited beetle*sex | 2 | 0.09 | 0.9543 |
| Treatment*population of cohabited beetle*sex | 2 | 0.96 | 0.6174 |
| Error | 415 |  |  |
